# The centrality of behavior change in health systems development

**DOI:** 10.9745/GHSP-D-13-00170

**Published:** 2014-02-11

**Authors:** Joseph F. Naimoli, Kathleen A. Parker, James Heiby

**Affiliations:** U.S. Agency for International Development, Washington, DC, USA, and Centers for Disease Control and Prevention, Atlanta, GA, USA.; Independent Consultant, Atlanta, GA, USA.; U.S. Agency for International Development, Washington, DC, USA.

The *Global Health: Science and Practice* editorial, “The 6 domains of behavior change: the missing health system building block,”^1^ serves as a welcome reminder of the pivotal role of behavior change in health systems development. Moreover, we agree that serious attention to health systems strengthening (HSS) and behavior change is necessary to achieve the ambitious Millennium Development Goals (MDGs) for health by 2015 and beyond. The editorial makes certain assumptions, however, about HSS and behavior change that we believe merit a response and clarification.

First, the editorial assumes that the absence of a “building block” on behavior change in the World Health Organization HSS framework^2^ represents a failure of the framework to adequately address the importance of behavior change in health systems development. Yet even a cursory review of the building blocks (governance, financing, service delivery, human resources, information, and medicines/vaccines/health technologies) reveals the crucial role of behavior change and the presence of behavior change interventions in each of the 6 subsystems represented by the framework. The targets of behavior change are not limited to individuals but include the full spectrum of health system actors, including client groups, communities, governments, nongovernmental organizations, and other entities.

For example, to increase transparency, accountability, and responsiveness to citizens, governments in low- and middle-income countries (LMICs) throughout the world are adopting new policy and regulatory interventions to alter the way they conduct business in the health sector to improve governance. In health financing, LMICs are using financial and nonfinancial incentives to encourage the adoption of health-promoting practices by providers, provider cooperatives, and consumers of health services. Many countries are adopting innovative health workforce and organizational change strategies to improve the delivery of health services that target multiple diseases. LMICs are increasingly recognizing that ensuring citizens' appropriate use of medicines, vaccines, and technologies is equally as important as guaranteeing their timely access to high-quality, efficacious products.

Second, by focusing solely on the building blocks framework, the editorial seems to imply, perhaps unintentionally, that professionals working to improve health systems—a diverse community that draws upon multiple social science disciplines—subscribe to a single approach or framework for HSS. Shakarishvili and colleagues have identified at least 11 different health systems frameworks that are in use by the global health community.^3^ We cite one example of an alternative framework that explicitly addresses behavior change and that has influenced the thinking of health systems experts and practitioners from LMICs for close to 2 decades.

Since the mid-1990s, the World Bank Institute, in collaboration with the Harvard School of Public Health, has been offering a “Flagship Course on Health Sector Reform and Sustainable Financing,” which uses an adaptable “control knobs” health systems framework.^4^ The control knobs are discrete areas of health system structure and function—financing, payment, organization, regulation, and behavior—which can be adjusted by various country actions to improve health system performance (access, coverage, efficiency, quality, and equity) to ultimately achieve long-term positive outcomes (health status, client satisfaction, and risk protection). The inclusion of a behavior control knob provides further evidence of health system analysts' acknowledgment of, and vigorous commitment to, the need for behavior change at all levels of the health system for continued growth and development. The control knobs framework reflects an emerging common theme, shared by many analysts, of a health system as a complex, dynamic whole: the constituent functional components of the health system are interconnected and interact to produce a range of effects, both positive and negative, intended and unintended.^5^^,^^6^

Third, the editorial's presentation of 6 domains of behavior, which include a mix of behaviors that influence, both positively and negatively, the health of individuals, communities, and the broader society, is a useful contribution. The subsequent list of bulleted “principles,” however, does not adequately reflect the systematic approach to, or address the complexities and challenges of, effective behavior change practice (from design to implementation to evaluation) for achievement of good health within any HSS framework. We recognize that an editorial cannot provide a full exposition of behavior change practice; nevertheless, we offer several caveats about practice as complementary information to ensure clarity.

Behavior change interventions must be tailored to the behavioral changes being sought, should be guided by behavior change theories,^7^ and will always have to be adapted to the particular situations in which they need to be considered, selected, and applied after careful assessment. Assessment requires, at a minimum, a clear articulation of the behaviors to be changed; an analysis of the characteristics of the individuals, groups, organizations, or populations that are the targets of change; and analysis of the factors influencing the behaviors. Assessment results enable scientists to identify and apply the appropriate theoretical framework(s) and the practical strategies most likely to achieve the desired outcomes.

In conclusion, frameworks for health systems strengthening are all serious, good-faith, albeit imperfect, efforts to understand complex dynamics. We would argue that most frameworks and health system practitioners recognize and embrace the importance of behavior change. The pressing challenge is to find and apply sufficient evidence and/or theory to justify the choice of the most cost-effective behavior change interventions that can be mounted, individually or together, in different settings, under different circumstances, to improve system performance and achieve the kinds of results that will improve people's health.
